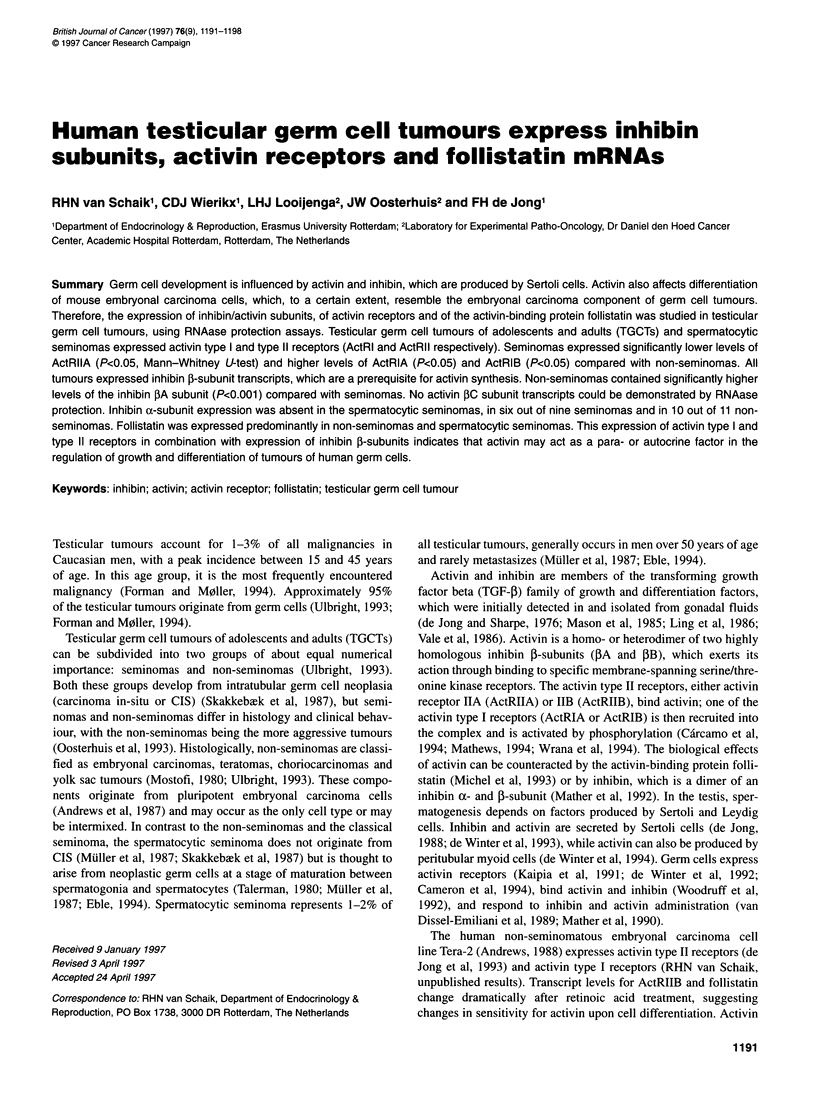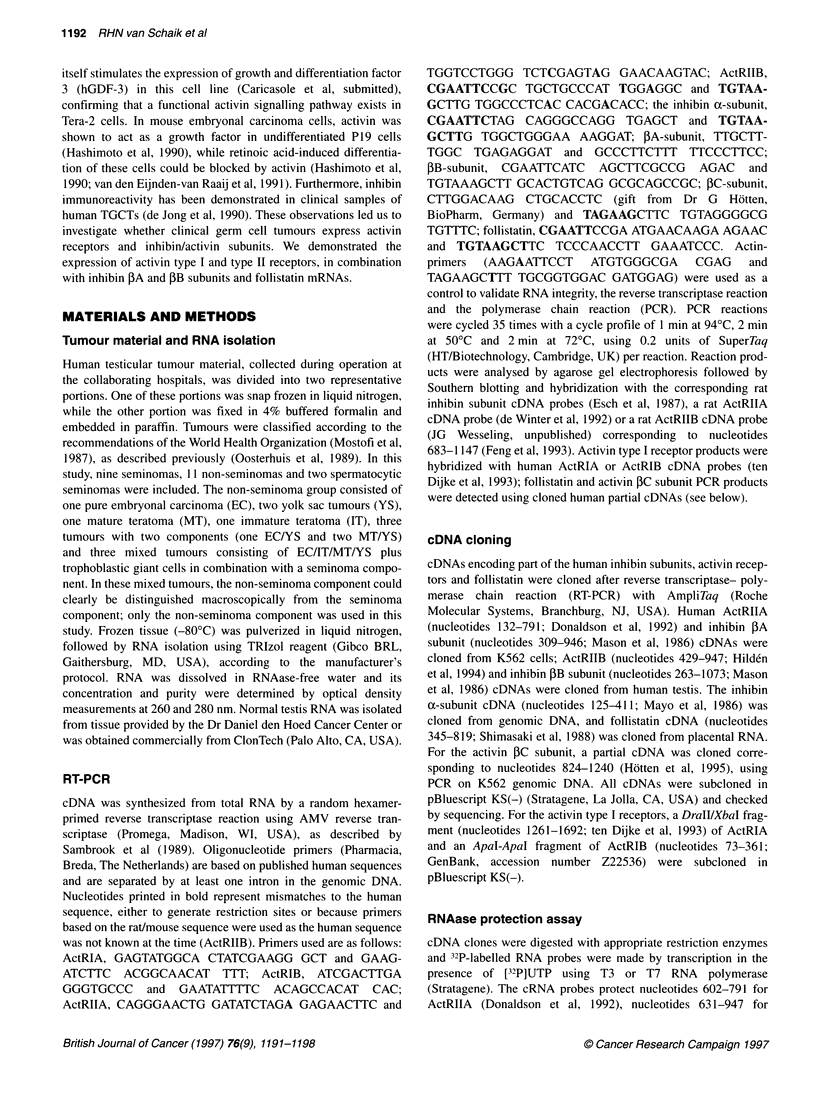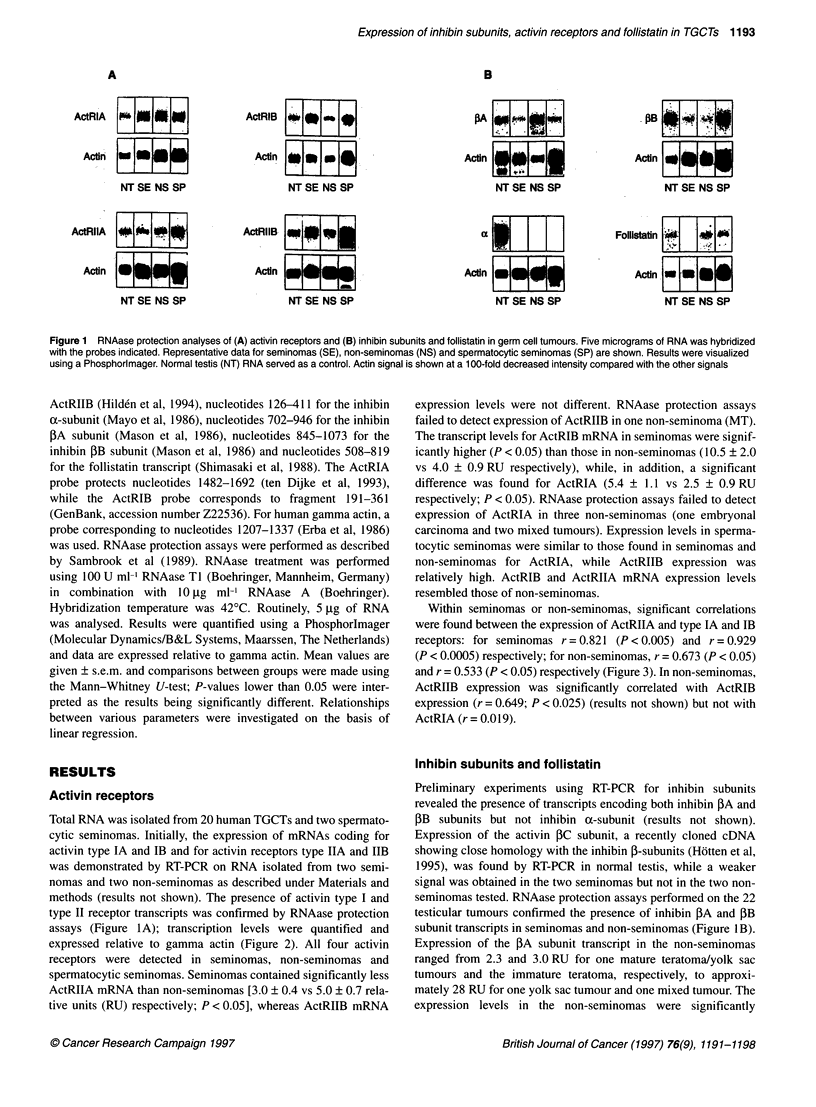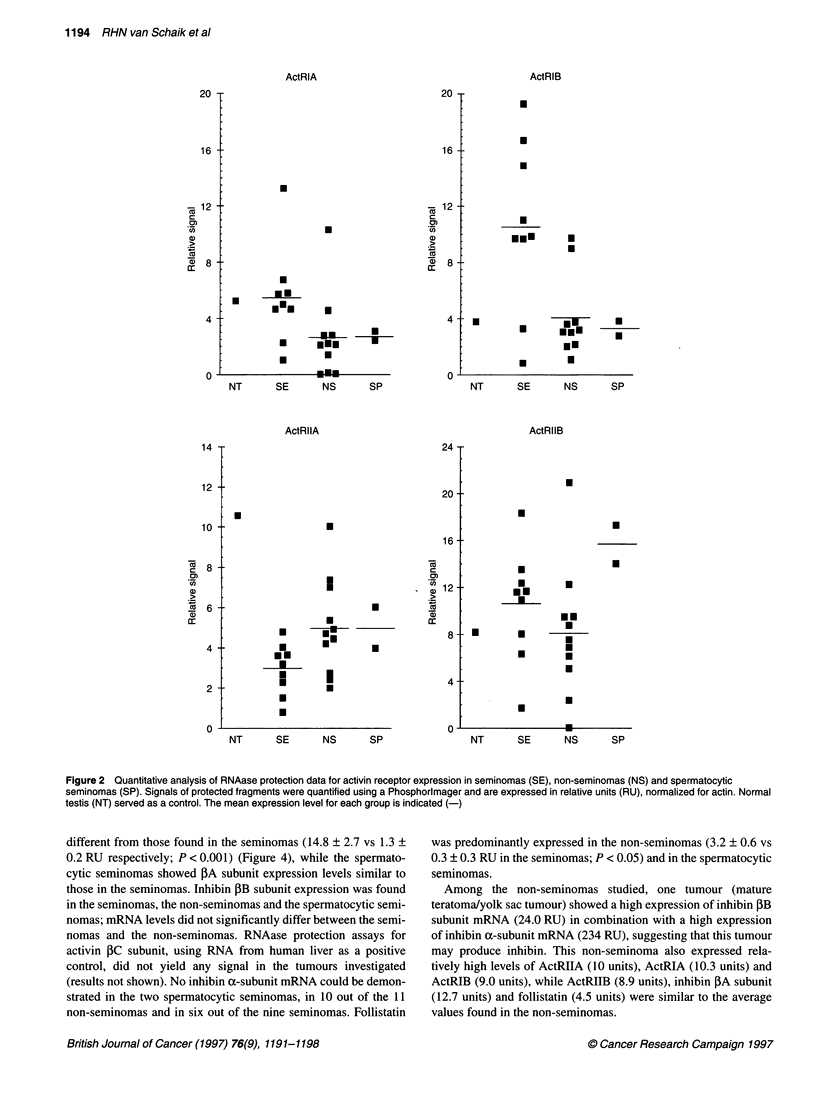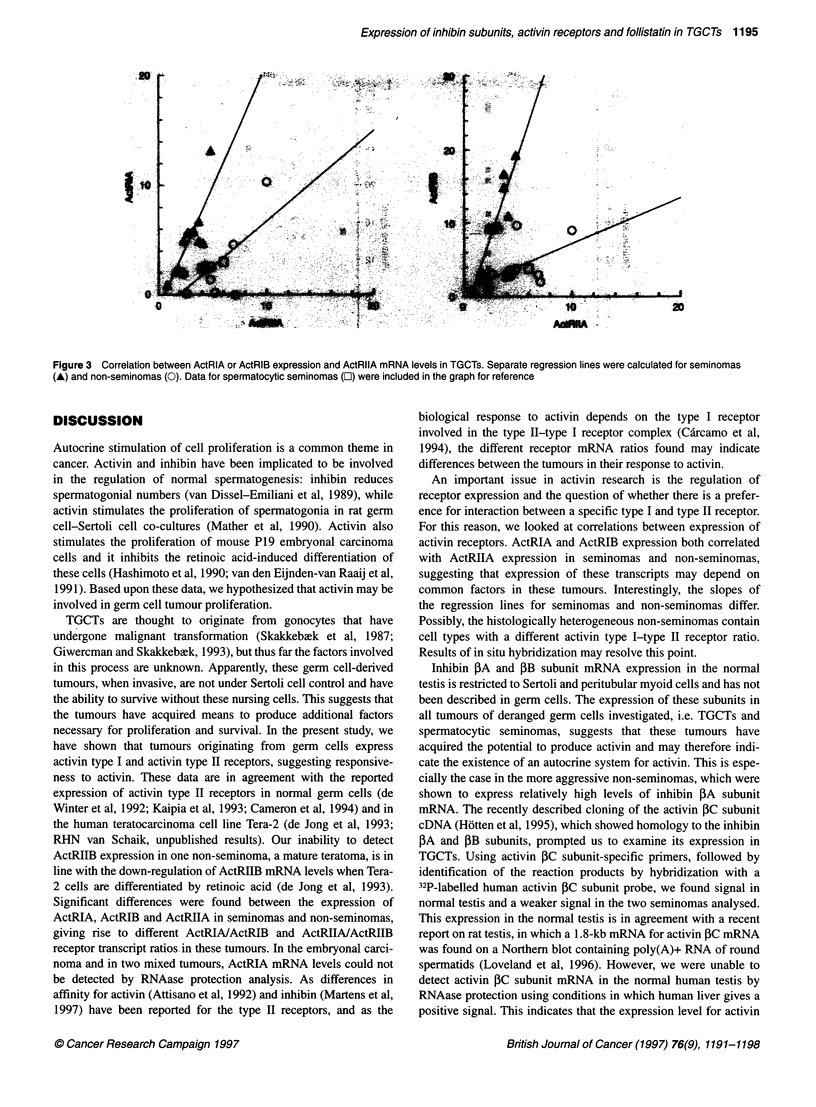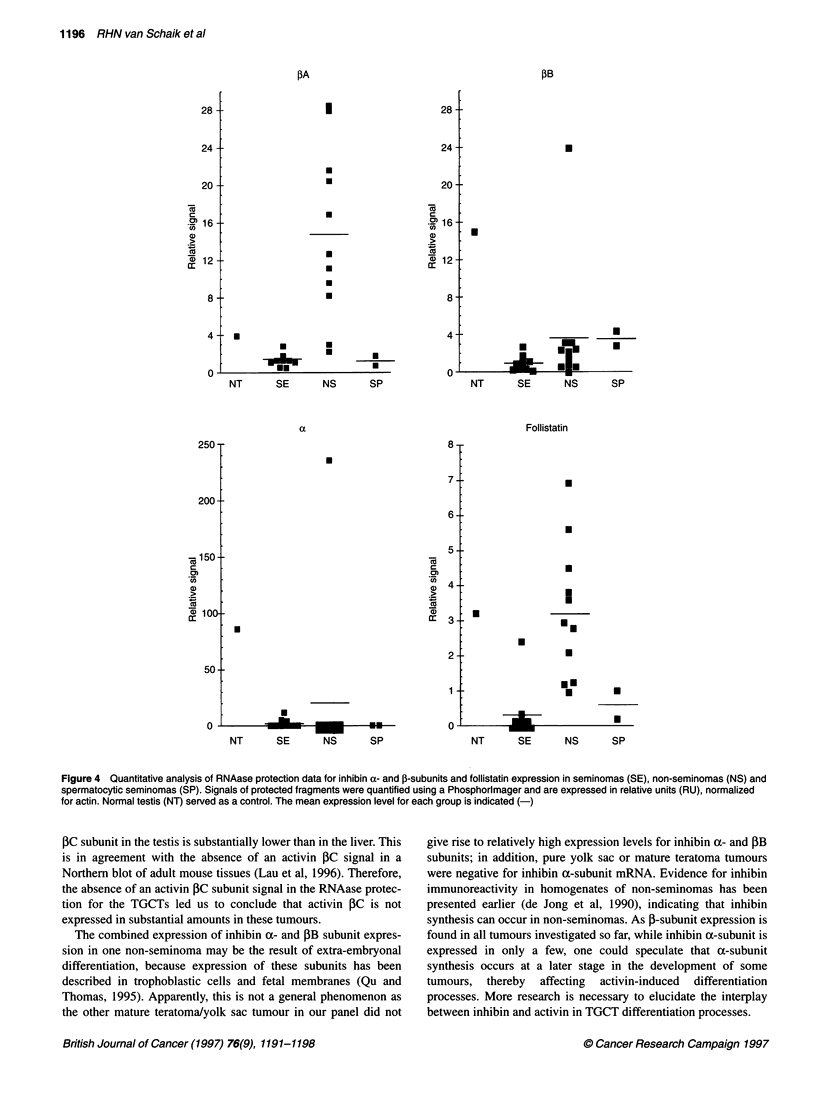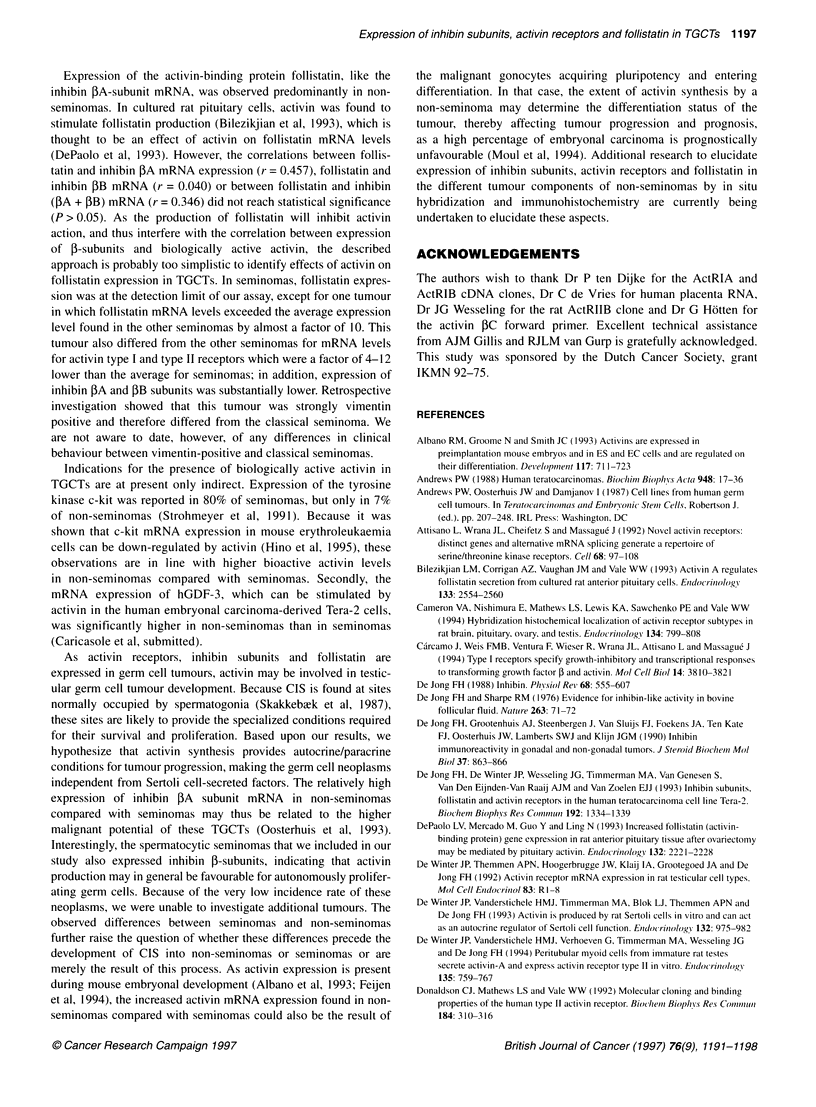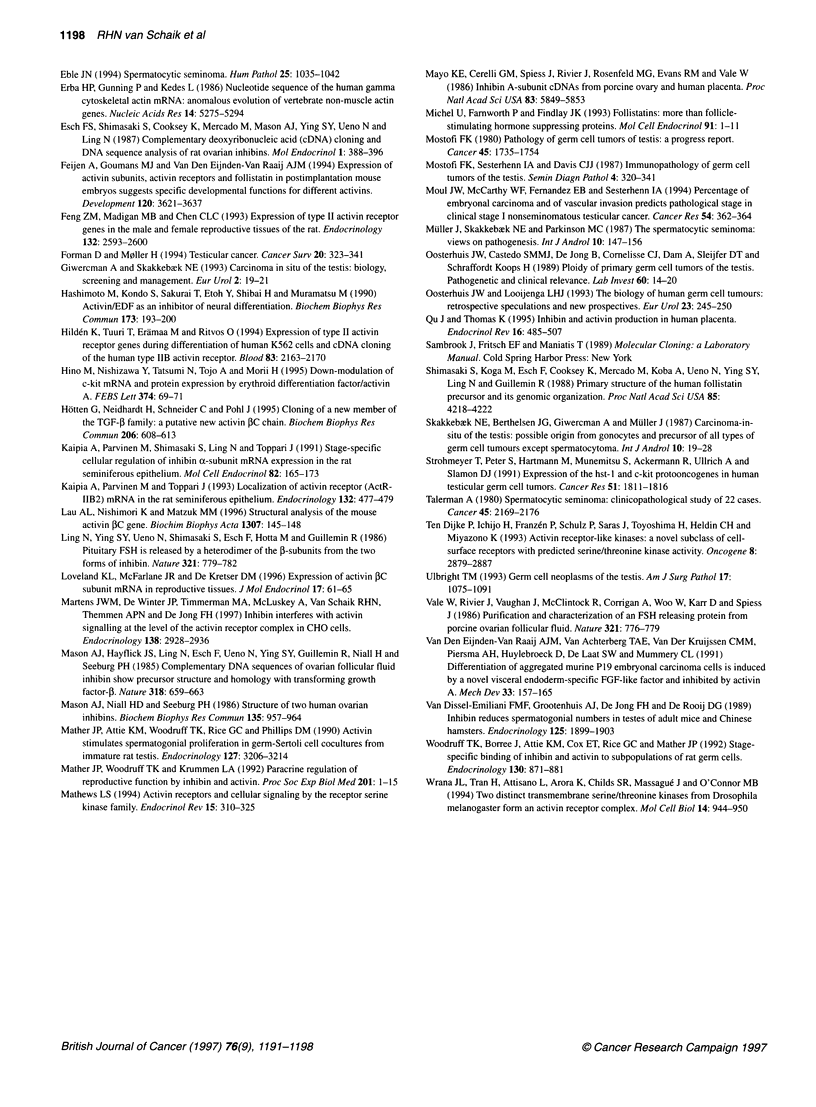# Human testicular germ cell tumours express inhibin subunits, activin receptors and follistatin mRNAs.

**DOI:** 10.1038/bjc.1997.532

**Published:** 1997

**Authors:** R. H. van Schaik, C. D. Wierikx, L. H. Looijenga, J. W. Oosterhuis, F. H. de Jong

**Affiliations:** Department of Endocrinology & Reproduction, Erasmus University Rotterdam, The Netherlands.

## Abstract

Germ cell development is influenced by activin and inhibin, which are produced by Sertoli cells. Activin also affects differentiation of mouse embryonal carcinoma cells, which, to a certain extent, resemble the embryonal carcinoma component of germ cell tumours. Therefore, the expression of inhibin/activin subunits, of activin receptors and of the activin-binding protein follistatin was studied in testicular germ cell tumours, using RNAase protection assays. Testicular germ cell tumours of adolescents and adults (TGCTs) and spermatocytic seminomas expressed activin type I and type II receptors (ActRI and ActRII respectively). Seminomas expressed significantly lower levels of ActRIIA (P<0.05, Mann-Whitney U-test) and higher levels of ActRIA (P<0.05) and ActRIB (P<0.05) compared with non-seminomas. All tumours expressed inhibin beta-subunit transcripts, which are a prerequisite for activin synthesis. Non-seminomas contained significantly higher levels of the inhibin betaA subunit (P<0.001) compared with seminomas. No activin betaC subunit transcripts could be demonstrated by RNAase protection. Inhibin alpha-subunit expression was absent in the spermatocytic seminomas, in six out of nine seminomas and in 10 out of 11 non-seminomas. Follistatin was expressed predominantly in non-seminomas and spermatocytic seminomas. This expression of activin type I and type II receptors in combination with expression of inhibin beta-subunits indicates that activin may act as a para- or autocrine factor in the regulation of growth and differentiation of tumours of human germ cells.


British Journal of Cancer (1997) 76(9), 1191–1198
© 1997 Cancer Research Campaign

### Human testicular germ cell tumours express inhibin subunits, activin receptors and follistatin mRNAs

**RHN van Schaik[1], CDJ Wierikx[1], LHJ Looijenga[2], JW Oosterhuis[2] and FH de Jong[1]**

[1]Department of Endocrinology & Reproduction, Erasmus University Rotterdam; [2]Laboratory for Experimental Patho-Oncology, Dr Daniel den Hoed Cancer Center, Academic Hospital Rotterdam, Rotterdam, The Netherlands

**Summary** Germ cell development is influenced by activin and inhibin, which are produced by Sertoli cells. Activin also affects differentiation of mouse embryonal carcinoma cells, which, to a certain extent, resemble the embryonal carcinoma component of germ cell tumours. Therefore, the expression of inhibin/activin subunits, of activin receptors and of the activin-binding protein follistatin was studied in testicular germ cell tumours, using RNAase protection assays. Testicular germ cell tumours of adolescents and adults (TGCTs) and spermatocytic seminomas expressed activin type I and type II receptors (ActRI and ActRII respectively). Seminomas expressed significantly lower levels of ActRIIA ($P$<0.05, Mann–Whitney $U$-test) and higher levels of ActRIA ($P$<0.05) and ActRIB ($P$<0.05) compared with non-seminomas. All tumours expressed inhibin β-subunit transcripts, which are a prerequisite for activin synthesis. Non-seminomas contained significantly higher levels of the inhibin βA subunit ($P$<0.001) compared with seminomas. No activin βC subunit transcripts could be demonstrated by RNAase protection. Inhibin α-subunit expression was absent in the spermatocytic seminomas, in six out of nine seminomas and in 10 out of 11 non-seminomas. Follistatin was expressed predominantly in non-seminomas and spermatocytic seminomas. This expression of activin type I and type II receptors in combination with expression of inhibin β-subunits indicates that activin may act as a para- or autocrine factor in the regulation of growth and differentiation of tumours of human germ cells.

**Keywords**: inhibin; activin; activin receptor; follistatin; testicular germ cell tumour

Testicular tumours account for 1–3% of all malignancies in Caucasian men, with a peak incidence between 15 and 45 years of age. In this age group, it is the most frequently encountered malignancy (Forman and Møller, 1994). Approximately 95% of the testicular tumours originate from germ cells (Ulbright, 1993; Forman and Møller, 1994).

Testicular germ cell tumours of adolescents and adults (TGCTs) can be subdivided into two groups of about equal numerical importance: seminomas and non-seminomas (Ulbright, 1993). Both these groups develop from intratubular germ cell neoplasia (carcinoma in-situ or CIS) (Skakkebæk et al, 1987), but seminomas and non-seminomas differ in histology and clinical behaviour, with the non-seminomas being the more aggressive tumours (Oosterhuis et al, 1993). Histologically, non-seminomas are classified as embryonal carcinomas, teratomas, choriocarcinomas and yolk sac tumours (Mostofi, 1980; Ulbright, 1993). These components originate from pluripotent embryonal carcinoma cells (Andrews et al, 1987) and may occur as the only cell type or may be intermixed. In contrast to the non-seminomas and the classical seminoma, the spermatocytic seminoma does not originate from CIS (Müller et al, 1987; Skakkebæk et al, 1987) but is thought to arise from neoplastic germ cells at a stage of maturation between spermatogonia and spermatocytes (Talerman, 1980; Müller et al, 1987; Eble, 1994). Spermatocytic seminoma represents 1–2% of all testicular tumours, generally occurs in men over 50 years of age and rarely metastasizes (Müller et al, 1987; Eble, 1994).

*Received 9 January 1997*
*Revised 3 April 1997*
*Accepted 24 April 1997*

*Correspondence to:* RHN van Schaik, Department of Endocrinology & Reproduction, PO Box 1738, 3000 DR Rotterdam, The Netherlands

Activin and inhibin are members of the transforming growth factor beta (TGF-β) family of growth and differentiation factors, which were initially detected in and isolated from gonadal fluids (de Jong and Sharpe, 1976; Mason et al, 1985; Ling et al, 1986; Vale et al, 1986). Activin is a homo- or heterodimer of two highly homologous inhibin β-subunits (βA and βB), which exerts its action through binding to specific membrane-spanning serine/threonine kinase receptors. The activin type II receptors, either activin receptor IIA (ActRIIA) or IIB (ActRIIB), bind activin; one of the activin type I receptors (ActRIA or ActRIB) is then recruited into the complex and is activated by phosphorylation (Cárcamo et al, 1994; Mathews, 1994; Wrana et al, 1994). The biological effects of activin can be counteracted by the activin-binding protein follistatin (Michel et al, 1993) or by inhibin, which is a dimer of an inhibin α- and β-subunit (Mather et al, 1992). In the testis, spermatogenesis depends on factors produced by Sertoli and Leydig cells. Inhibin and activin are secreted by Sertoli cells (de Jong, 1988; de Winter et al, 1993), while activin can also be produced by peritubular myoid cells (de Winter et al, 1994). Germ cells express activin receptors (Kaipia et al, 1991; de Winter et al, 1992; Cameron et al, 1994), bind activin and inhibin (Woodruff et al, 1992), and respond to inhibin and activin administration (van Dissel-Emiliani et al, 1989; Mather et al, 1990).

The human non-seminomatous embryonal carcinoma cell line Tera-2 (Andrews, 1988) expresses activin type II receptors (de Jong et al, 1993) and activin type I receptors (RHN van Schaik, unpublished results). Transcript levels for ActRIIB and follistatin change dramatically after retinoic acid treatment, suggesting changes in sensitivity for activin upon cell differentiation. Activin itself stimulates the expression of growth and differentiation factor 3 (hGDF-3) in this cell line (Caricasole et al, submitted), confirming that a functional activin signalling pathway exists in Tera-2 cells. In mouse embryonal carcinoma cells, activin was shown to act as a growth factor in undifferentiated P19 cells (Hashimoto et al, 1990), while retinoic acid-induced differentiation of these cells could be blocked by activin (Hashimoto et al, 1990; van den Eijnden-van Raaij et al, 1991). Furthermore, inhibin immunoreactivity has been demonstrated in clinical samples of human TGCTs (de Jong et al, 1990). These observations led us to investigate whether clinical germ cell tumours express activin receptors and inhibin/activin subunits. We demonstrated the expression of activin type I and type II receptors, in combination with inhibin βA and βB subunits and follistatin mRNAs.

### MATERIALS AND METHODS

### Tumour material and RNA isolation

Human testicular tumour material, collected during operation at the collaborating hospitals, was divided into two representative portions. One of these portions was snap frozen in liquid nitrogen, while the other portion was fixed in 4% buffered formalin and embedded in paraffin. Tumours were classified according to the recommendations of the World Health Organization (Mostofi et al, 1987), as described previously (Oosterhuis et al, 1989). In this study, nine seminomas, 11 non-seminomas and two spermatocytic seminomas were included. The non-seminoma group consisted of one pure embryonal carcinoma (EC), two yolk sac tumours (YS), one mature teratoma (MT), one immature teratoma (IT), three tumours with two components (one EC/YS and two MT/YS) and three mixed tumours consisting of EC/IT/MT/YS plus trophoblastic giant cells in combination with a seminoma component. In these mixed tumours, the non-seminoma component could clearly be distinguished macroscopically from the seminoma component; only the non-seminoma component was used in this study. Frozen tissue (–80°C) was pulverized in liquid nitrogen, followed by RNA isolation using TRIzol reagent (Gibco BRL, Gaithersburg, MD, USA), according to the manufacturer's protocol. RNA was dissolved in RNAase-free water and its concentration and purity were determined by optical density measurements at 260 and 280 nm. Normal testis RNA was isolated from tissue provided by the Dr Daniel den Hoed Cancer Center or was obtained commercially from ClonTech (Palo Alto, CA, USA).

### RT-PCR

cDNA was synthesized from total RNA by a random hexamer-primed reverse transcriptase reaction using AMV reverse transcriptase (Promega, Madison, WI, USA), as described by Sambrook et al (1989). Oligonucleotide primers (Pharmacia, Breda, The Netherlands) are based on published human sequences and are separated by at least one intron in the genomic DNA. Nucleotides printed in bold represent mismatches to the human sequence, either to generate restriction sites or because primers based on the rat/mouse sequence were used as the human sequence was not known at the time (ActRIIB). Primers used are as follows: ActRIA, GAGTATGGCA CTATCGAAGG GCT and GAAGATCTTC ACGGCAACAT TTT; ActRIB, ATCGACTTGA GGGTGCCC and GAATATTTTC ACAGCCACAT CAC; ActRIIA, CAGGGAACTG GATATCTAGA GAGAACTTC and TGGTCCTGGG TCTCGAGTAG GAACAAGTAC; ActRIIB, **CGAATTCCGC** TGCTGCCCAT **TGGAGGC** and **TGTAAGCTTG** TGGCCCTCAC CACGACACC; the inhibin α-subunit, **CGAATTC**TAG CAGGGCCAGG TGAGCT and **TGTAAGCTTG** TGGCTGGGAA AAGGAT; βA-subunit, TTGCTTTGGC TGAGAGGAT and GCCCTTCTTT TTCCCTTCC; βB-subunit, CGAATTCATC AGCTTCGCCG AGAC and TGTAAAGCTT GCACTGTCAG GCGCAGCCGC; βC-subunit, CTTGGACAAG CTGCACCTC (gift from Dr G Hötten, BioPharm, Germany) and **TAGAAG**CTTC TGTAGGGGCG TGTTTC; follistatin, **CGAATT**CCGA ATGAACAAGA AGAAC and **TGTAAGCT**TC TCCCAACCTT GAAATCCC. Actin-primers (AAGAATTCCT ATGTGGGCGA CGAG and TAGAAGCTTT TGCGGTGGAC GATGGAG) were used as a control to validate RNA integrity, the reverse transcriptase reaction and the polymerase chain reaction (PCR). PCR reactions were cycled 35 times with a cycle profile of 1 min at 94°C, 2 min at 50°C and 2 min at 72°C, using 0.2 units of Super*Taq* (HT/Biotechnology, Cambridge, UK) per reaction. Reaction products were analysed by agarose gel electrophoresis followed by Southern blotting and hybridization with the corresponding rat inhibin subunit cDNA probes (Esch et al, 1987), a rat ActRIIA cDNA probe (de Winter et al, 1992) or a rat ActRIIB cDNA probe (JG Wesseling, unpublished) corresponding to nucleotides 683–1147 (Feng et al, 1993). Activin type I receptor products were hybridized with human ActRIA or ActRIB cDNA probes (ten Dijke et al, 1993); follistatin and activin βC subunit PCR products were detected using cloned human partial cDNAs (see below).

### cDNA cloning cDNAs encoding part of the human inhibin subunits, activin receptors and follistatin were cloned after reverse transcriptase– polymerase chain reaction (RT-PCR) with Ampli*Taq* (Roche Molecular Systems, Branchburg, NJ, USA). Human ActRIIA (nucleotides 132–791; Donaldson et al, 1992) and inhibin βA subunit (nucleotides 309–946; Mason et al, 1986) cDNAs were cloned from K562 cells; ActRIIB (nucleotides 429–947; Hildén et al, 1994) and inhibin βB subunit (nucleotides 263–1073; Mason et al, 1986) cDNAs were cloned from human testis. The inhibin α-subunit cDNA (nucleotides 125–411; Mayo et al, 1986) was cloned from genomic DNA, and follistatin cDNA (nucleotides 345–819; Shimasaki et al, 1988) was cloned from placental RNA. For the activin βC subunit, a partial cDNA was cloned corresponding to nucleotides 824–1240 (Hötten et al, 1995), using PCR on K562 genomic DNA. All cDNAs were subcloned in pBluescript KS(–) (Stratagene, La Jolla, CA, USA) and checked by sequencing. For the activin type I receptors, a *Dra*II/*Xba*I fragment (nucleotides 1261–1692; ten Dijke et al, 1993) of ActRIA and an *Apa*I-*Apa*I fragment of ActRIB (nucleotides 73–361; GenBank, accession number Z22536) were subcloned in pBluescript KS(–).

### RNAase protection assay cDNA clones were digested with appropriate restriction enzymes and $^{32}$P-labelled RNA probes were made by transcription in the presence of [$^{32}$P]UTP using T3 or T7 RNA polymerase (Stratagene). The cRNA probes protect nucleotides 602–791 for ActRIIA (Donaldson et al, 1992), nucleotides 631–947 for

© Cancer Research Campaign 1997

**Figure 1** RNAase protection analyses of (**A**) activin receptors and (**B**) inhibin subunits and follistatin in germ cell tumours. Five micrograms of RNA was hybridized with the probes indicated. Representative data for seminomas (SE), non-seminomas (NS) and spermatocytic seminomas (SP) are shown. Results were visualized using a PhosphorImager. Normal testis (NT) RNA served as a control. Actin signal is shown at a 100-fold decreased intensity compared with the other signals

ActRIIB (Hildén et al, 1994), nucleotides 126–411 for the inhibin α-subunit (Mayo et al, 1986), nucleotides 702–946 for the inhibin βA subunit (Mason et al, 1986), nucleotides 845–1073 for the inhibin βB subunit (Mason et al, 1986) and nucleotides 508–819 for the follistatin transcript (Shimasaki et al, 1988). The ActRIA probe protects nucleotides 1482–1692 (ten Dijke et al, 1993), while the ActRIB probe corresponds to fragment 191–361 (GenBank, accession number Z22536). For human gamma actin, a probe corresponding to nucleotides 1207–1337 (Erba et al, 1986) was used. RNAase protection assays were performed as described by Sambrook et al (1989). RNAase treatment was performed using 100 U $ml^{-1}$ RNAase T1 (Boehringer, Mannheim, Germany) in combination with 10 μg $ml^{-1}$ RNAase A (Boehringer). Hybridization temperature was 42°C. Routinely, 5 μg of RNA was analysed. Results were quantified using a PhosphorImager (Molecular Dynamics/B&L Systems, Maarssen, The Netherlands) and data are expressed relative to gamma actin. Mean values are given ± s.e.m. and comparisons between groups were made using the Mann–Whitney *U*-test; *P*-values lower than 0.05 were interpreted as the results being significantly different. Relationships between various parameters were investigated on the basis of linear regression.

### RESULTS

### Activin receptors

Total RNA was isolated from 20 human TGCTs and two spermatocytic seminomas. Initially, the expression of mRNAs coding for activin type IA and IB and for activin receptors type IIA and IIB was demonstrated by RT-PCR on RNA isolated from two seminomas and two non-seminomas as described under Materials and methods (results not shown). The presence of activin type I and type II receptor transcripts was confirmed by RNAase protection assays (Figure 1A); transcription levels were quantified and expressed relative to gamma actin (Figure 2). All four activin receptors were detected in seminomas, non-seminomas and spermatocytic seminomas. Seminomas contained significantly less ActRIIA mRNA than non-seminomas [3.0 ± 0.4 vs 5.0 ± 0.7 relative units (RU) respectively; $P < 0.05$], whereas ActRIIB mRNA expression levels were not different. RNAase protection assays failed to detect expression of ActRIIB in one non-seminoma (MT). The transcript levels for ActRIB mRNA in seminomas were significantly higher ($P < 0.05$) than those in non-seminomas (10.5 ± 2.0 vs 4.0 ± 0.9 RU respectively), while, in addition, a significant difference was found for ActRIA (5.4 ± 1.1 vs 2.5 ± 0.9 RU respectively; $P < 0.05$). RNAase protection assays failed to detect expression of ActRIA in three non-seminomas (one embryonal carcinoma and two mixed tumours). Expression levels in spermatocytic seminomas were similar to those found in seminomas and non-seminomas for ActRIA, while ActRIIB expression was relatively high. ActRIB and ActRIIA mRNA expression levels resembled those of non-seminomas.

Within seminomas or non-seminomas, significant correlations were found between the expression of ActRIIA and type IA and IB receptors: for seminomas $r = 0.821$ ($P < 0.005$) and $r = 0.929$ ($P < 0.0005$) respectively; for non-seminomas, $r = 0.673$ ($P < 0.05$) and $r = 0.533$ ($P < 0.05$) respectively (Figure 3). In non-seminomas, ActRIIB expression was significantly correlated with ActRIB expression ($r = 0.649$; $P < 0.025$) (results not shown) but not with ActRIA ($r = 0.019$).

### Inhibin subunits and follistatin

Preliminary experiments using RT-PCR for inhibin subunits revealed the presence of transcripts encoding both inhibin βA and βB subunits but not inhibin α-subunit (results not shown). Expression of the activin βC subunit, a recently cloned cDNA showing close homology with the inhibin β-subunits (Hötten et al, 1995), was found by RT-PCR in normal testis, while a weaker signal was obtained in the two seminomas but not in the two non-seminomas tested. RNAase protection assays performed on the 22 testicular tumours confirmed the presence of inhibin βA and βB subunit transcripts in seminomas and non-seminomas (Figure 1B). Expression of the βA subunit transcript in the non-seminomas ranged from 2.3 and 3.0 RU for one mature teratoma/yolk sac tumours and the immature teratoma, respectively, to approximately 28 RU for one yolk sac tumour and one mixed tumour. The expression levels in the non-seminomas were significantly

© Cancer Research Campaign 1997

**Figure 2** Quantitative analysis of RNAase protection data for activin receptor expression in seminomas (SE), non-seminomas (NS) and spermatocytic seminomas (SP). Signals of protected fragments were quantified using a PhosphorImager and are expressed in relative units (RU), normalized for actin. Normal testis (NT) served as a control. The mean expression level for each group is indicated (—)

different from those found in the seminomas (14.8 ± 2.7 vs 1.3 ± 0.2 RU respectively; $P < 0.001$) (Figure 4), while the spermatocytic seminomas showed βA subunit expression levels similar to those in the seminomas. Inhibin βB subunit expression was found in the seminomas, the non-seminomas and the spermatocytic seminomas; mRNA levels did not significantly differ between the seminomas and the non-seminomas. RNAase protection assays for activin βC subunit, using RNA from human liver as a positive control, did not yield any signal in the tumours investigated (results not shown). No inhibin α-subunit mRNA could be demonstrated in the two spermatocytic seminomas, in 10 out of the 11 non-seminomas and in six out of the nine seminomas. Follistatin was predominantly expressed in the non-seminomas (3.2 ± 0.6 vs 0.3 ± 0.3 RU in the seminomas; $P < 0.05$) and in the spermatocytic seminomas.

Among the non-seminomas studied, one tumour (mature teratoma/yolk sac tumour) showed a high expression of inhibin βB subunit mRNA (24.0 RU) in combination with a high expression of inhibin α-subunit mRNA (234 RU), suggesting that this tumour may produce inhibin. This non-seminoma also expressed relatively high levels of ActRIIA (10 units), ActRIA (10.3 units) and ActRIB (9.0 units), while ActRIIB (8.9 units), inhibin βA subunit (12.7 units) and follistatin (4.5 units) were similar to the average values found in the non-seminomas.

© Cancer Research Campaign 1997

**Figure 3** Correlation between ActRIA or ActRIB expression and ActRIIA mRNA levels in TGCTs. Separate regression lines were calculated for seminomas (▲) and non-seminomas (○). Data for spermatocytic seminomas (□) were included in the graph for reference

### DISCUSSION

Autocrine stimulation of cell proliferation is a common theme in cancer. Activin and inhibin have been implicated to be involved in the regulation of normal spermatogenesis: inhibin reduces spermatogonial numbers (van Dissel–Emiliani et al, 1989), while activin stimulates the proliferation of spermatogonia in rat germ cell–Sertoli cell co-cultures (Mather et al, 1990). Activin also stimulates the proliferation of mouse P19 embryonal carcinoma cells and it inhibits the retinoic acid-induced differentiation of these cells (Hashimoto et al, 1990; van den Eijnden-van Raaij et al, 1991). Based upon these data, we hypothesized that activin may be involved in germ cell tumour proliferation.

TGCTs are thought to originate from gonocytes that have undergone malignant transformation (Skakkebæk et al, 1987; Giwercman and Skakkebæk, 1993), but thus far the factors involved in this process are unknown. Apparently, these germ cell-derived tumours, when invasive, are not under Sertoli cell control and have the ability to survive without these nursing cells. This suggests that the tumours have acquired means to produce additional factors necessary for proliferation and survival. In the present study, we have shown that tumours originating from germ cells express activin type I and activin type II receptors, suggesting responsiveness to activin. These data are in agreement with the reported expression of activin type II receptors in normal germ cells (de Winter et al, 1992; Kaipia et al, 1993; Cameron et al, 1994) and in the human teratocarcinoma cell line Tera-2 (de Jong et al, 1993; RHN van Schaik, unpublished results). Our inability to detect ActRIIB expression in one non-seminoma, a mature teratoma, is in line with the down-regulation of ActRIIB mRNA levels when Tera-2 cells are differentiated by retinoic acid (de Jong et al, 1993). Significant differences were found between the expression of ActRIA, ActRIB and ActRIIA in seminomas and non-seminomas, giving rise to different ActRIA/ActRIB and ActRIIA/ActRIIB receptor transcript ratios in these tumours. In the embryonal carcinoma and in two mixed tumours, ActRIA mRNA levels could not be detected by RNAase protection analysis. As differences in affinity for activin (Attisano et al, 1992) and inhibin (Martens et al, 1997) have been reported for the type II receptors, and as the biological response to activin depends on the type I receptor involved in the type II–type I receptor complex (Cárcamo et al, 1994), the different receptor mRNA ratios found may indicate differences between the tumours in their response to activin.

An important issue in activin research is the regulation of receptor expression and the question of whether there is a preference for interaction between a specific type I and type II receptor. For this reason, we looked at correlations between expression of activin receptors. ActRIA and ActRIB expression both correlated with ActRIIA expression in seminomas and non-seminomas, suggesting that expression of these transcripts may depend on common factors in these tumours. Interestingly, the slopes of the regression lines for seminomas and non-seminomas differ. Possibly, the histologically heterogeneous non-seminomas contain cell types with a different activin type I–type II receptor ratio. Results of in situ hybridization may resolve this point.

Inhibin βA and βB subunit mRNA expression in the normal testis is restricted to Sertoli and peritubular myoid cells and has not been described in germ cells. The expression of these subunits in all tumours of deranged germ cells investigated, i.e. TGCTs and spermatocytic seminomas, suggests that these tumours have acquired the potential to produce activin and may therefore indicate the existence of an autocrine system for activin. This is especially the case in the more aggressive non-seminomas, which were shown to express relatively high levels of inhibin βA subunit mRNA. The recently described cloning of the activin βC subunit cDNA (Hötten et al, 1995), which showed homology to the inhibin βA and βB subunits, prompted us to examine its expression in TGCTs. Using activin βC subunit-specific primers, followed by identification of the reaction products by hybridization with a $^{32}$P-labelled human activin βC subunit probe, we found signal in normal testis and a weaker signal in the two seminomas analysed. This expression in the normal testis is in agreement with a recent report on rat testis, in which a 1.8-kb mRNA for activin βC mRNA was found on a Northern blot containing poly(A)+ RNA of round spermatids (Loveland et al, 1996). However, we were unable to detect activin βC subunit mRNA in the normal human testis by RNAase protection using conditions in which human liver gives a positive signal. This indicates that the expression level for activin

*© Cancer Research Campaign 1997* 

**Figure 4** Quantitative analysis of RNAase protection data for inhibin α- and β-subunits and follistatin expression in seminomas (SE), non-seminomas (NS) and spermatocytic seminomas (SP). Signals of protected fragments were quantified using a PhosphorImager and are expressed in relative units (RU), normalized for actin. Normal testis (NT) served as a control. The mean expression level for each group is indicated (—)

βC subunit in the testis is substantially lower than in the liver. This is in agreement with the absence of an activin βC signal in a Northern blot of adult mouse tissues (Lau et al, 1996). Therefore, the absence of an activin βC subunit signal in the RNAase protection for the TGCTs led us to conclude that activin βC is not expressed in substantial amounts in these tumours.

The combined expression of inhibin α- and βB subunit expression in one non-seminoma may be the result of extra-embryonal differentiation, because expression of these subunits has been described in trophoblastic cells and fetal membranes (Qu and Thomas, 1995). Apparently, this is not a general phenomenon as the other mature teratoma/yolk sac tumour in our panel did not give rise to relatively high expression levels for inhibin α- and βB subunits; in addition, pure yolk sac or mature teratoma tumours were negative for inhibin α-subunit mRNA. Evidence for inhibin immunoreactivity in homogenates of non-seminomas has been presented earlier (de Jong et al, 1990), indicating that inhibin synthesis can occur in non-seminomas. As β-subunit expression is found in all tumours investigated so far, while inhibin α-subunit is expressed in only a few, one could speculate that α-subunit synthesis occurs at a later stage in the development of some tumours, thereby affecting activin-induced differentiation processes. More research is necessary to elucidate the interplay between inhibin and activin in TGCT differentiation processes.

© Cancer Research Campaign 1997

Expression of the activin-binding protein follistatin, like the inhibin βA-subunit mRNA, was observed predominantly in non-seminomas. In cultured rat pituitary cells, activin was found to stimulate follistatin production (Bilezikjian et al, 1993), which is thought to be an effect of activin on follistatin mRNA levels (DePaolo et al, 1993). However, the correlations between follistatin and inhibin βA mRNA expression ($r = 0.457$), follistatin and inhibin βB mRNA ($r = 0.040$) or between follistatin and inhibin (βA + βB) mRNA ($r = 0.346$) did not reach statistical significance ($P > 0.05$). As the production of follistatin will inhibit activin action, and thus interfere with the correlation between expression of β-subunits and biologically active activin, the described approach is probably too simplistic to identify effects of activin on follistatin expression in TGCTs. In seminomas, follistatin expression was at the detection limit of our assay, except for one tumour in which follistatin mRNA levels exceeded the average expression level found in the other seminomas by almost a factor of 10. This tumour also differed from the other seminomas for mRNA levels for activin type I and type II receptors which were a factor of 4–12 lower than the average for seminomas; in addition, expression of inhibin βA and βB subunits was substantially lower. Retrospective investigation showed that this tumour was strongly vimentin positive and therefore differed from the classical seminoma. We are not aware to date, however, of any differences in clinical behaviour between vimentin-positive and classical seminomas.

Indications for the presence of biologically active activin in TGCTs are at present only indirect. Expression of the tyrosine kinase c-kit was reported in 80% of seminomas, but only in 7% of non-seminomas (Strohmeyer et al, 1991). Because it was shown that c-kit mRNA expression in mouse erythroleukaemia cells can be down-regulated by activin (Hino et al, 1995), these observations are in line with higher bioactive activin levels in non-seminomas compared with seminomas. Secondly, the mRNA expression of hGDF-3, which can be stimulated by activin in the human embryonal carcinoma-derived Tera-2 cells, was significantly higher in non-seminomas than in seminomas (Caricasole et al, submitted).

As activin receptors, inhibin subunits and follistatin are expressed in germ cell tumours, activin may be involved in testicular germ cell tumour development. Because CIS is found at sites normally occupied by spermatogonia (Skakkebæk et al, 1987), these sites are likely to provide the specialized conditions required for their survival and proliferation. Based upon our results, we hypothesize that activin synthesis provides autocrine/paracrine conditions for tumour progression, making the germ cell neoplasms independent from Sertoli cell-secreted factors. The relatively high expression of inhibin βA subunit mRNA in non-seminomas compared with seminomas may thus be related to the higher malignant potential of these TGCTs (Oosterhuis et al, 1993). Interestingly, the spermatocytic seminomas that we included in our study also expressed inhibin β-subunits, indicating that activin production may in general be favourable for autonomously proliferating germ cells. Because of the very low incidence rate of these neoplasms, we were unable to investigate additional tumours. The observed differences between seminomas and non-seminomas further raise the question of whether these differences precede the development of CIS into non-seminomas or seminomas or are merely the result of this process. As activin expression is present during mouse embryonal development (Albano et al, 1993; Feijen et al, 1994), the increased activin mRNA expression found in non-seminomas compared with seminomas could also be the result of the malignant gonocytes acquiring pluripotency and entering differentiation. In that case, the extent of activin synthesis by a non-seminoma may determine the differentiation status of the tumour, thereby affecting tumour progression and prognosis, as a high percentage of embryonal carcinoma is prognostically unfavourable (Moul et al, 1994). Additional research to elucidate expression of inhibin subunits, activin receptors and follistatin in the different tumour components of non-seminomas by in situ hybridization and immunohistochemistry are currently being undertaken to elucidate these aspects.

### ACKNOWLEDGEMENTS

The authors wish to thank Dr P ten Dijke for the ActRIA and ActRIB cDNA clones, Dr C de Vries for human placenta RNA, Dr JG Wesseling for the rat ActRIIB clone and Dr G Hötten for the activin βC forward primer. Excellent technical assistance from AJM Gillis and RJLM van Gurp is gratefully acknowledged. This study was sponsored by the Dutch Cancer Society, grant IKMN 92–75.

### REFERENCES

Albano RM, Groome N and Smith JC (1993) Activins are expressed in preimplantation mouse embryos and in ES and EC cells and are regulated on their differentiation. *Development* **117**: 711–723

Andrews PW (1988) Human teratocarcinomas. *Biochim Biophys Acta* **948**: 17–36

Andrews PW, Oosterhuis JW and Damjanov I (1987) Cell lines from human germ cell tumours. In *Teratocarcinomas and Embryonic Stem Cells*, Robertson J. (ed.), pp. 207–248. IRL Press: Washington, DC

Attisano L, Wrana JL, Cheifetz S and Massagué J (1992) Novel activin receptors: distinct genes and alternative mRNA splicing generate a repertoire of serine/threonine kinase receptors. *Cell* **68**: 97–108

Bilezikjian LM, Corrigan AZ, Vaughan JM and Vale WW (1993) Activin A regulates follistatin secretion from cultured rat anterior pituitary cells. *Endocrinology* **133**: 2554–2560

Cameron VA, Nishimura E, Mathews LS, Lewis KA, Sawchenko PE and Vale WW (1994) Hybridization histochemical localization of activin receptor subtypes in rat brain, pituitary, ovary, and testis. *Endocrinology* **134**: 799–808

Cárcamo J, Weis FMB, Ventura F, Wieser R, Wrana JL, Attisano L and Massagué J (1994) Type I receptors specify growth-inhibitory and transcriptional responses to transforming growth factor β and activin. *Mol Cell Biol* **14**: 3810–3821

De Jong FH (1988) Inhibin. *Physiol Rev* **68**: 555–607

De Jong FH and Sharpe RM (1976) Evidence for inhibin-like activity in bovine follicular fluid. *Nature* **263**: 71–72

De Jong FH, Grootenhuis AJ, Steenbergen J, Van Sluijs FJ, Foekens JA, Ten Kate FJ, Oosterhuis JW, Lamberts SWJ and Klijn JGM (1990) Inhibin immunoreactivity in gonadal and non-gonadal tumors. *J Steroid Biochem Mol Biol* **37**: 863–866

De Jong FH, De Winter JP, Wesseling JG, Timmerman MA, Van Genesen S, Van Den Eijnden-Van Raaij AJM and Van Zoelen EJJ (1993) Inhibin subunits, follistatin and activin receptors in the human teratocarcinoma cell line Tera-2. *Biochem Biophys Res Commun* **192**: 1334–1339

DePaolo LV, Mercado M, Guo Y and Ling N (1993) Increased follistatin (activin-binding protein) gene expression in rat anterior pituitary tissue after ovariectomy may be mediated by pituitary activin. *Endocrinology* **132**: 2221–2228

De Winter JP, Themmen APN, Hoogerbrugge JW, Klaij IA, Grootegoed JA and De Jong FH (1992) Activin receptor mRNA expression in rat testicular cell types. *Mol Cell Endocrinol* **83**: R1–8

De Winter JP, Vanderstichele HMJ, Timmerman MA, Blok LJ, Themmen APN and De Jong FH (1993) Activin is produced by rat Sertoli cells in vitro and can act as an autocrine regulator of Sertoli cell function. *Endocrinology* **132**: 975–982

De Winter JP, Vanderstichele HMJ, Verhoeven G, Timmerman MA, Wesseling JG and De Jong FH (1994) Peritubular myoid cells from immature rat testes secrete activin-A and express activin receptor type II in vitro. *Endocrinology* **135**: 759–767

Donaldson CJ, Mathews LS and Vale WW (1992) Molecular cloning and binding properties of the human type II activin receptor. *Biochem Biophys Res Commun* **184**: 310–316

© Cancer Research Campaign 1997

Eble JN (1994) Spermatocytic seminoma. *Hum Pathol* **25**: 1035–1042
Erba HP, Gunning P and Kedes L (1986) Nucleotide sequence of the human gamma cytoskeletal actin mRNA: anomalous evolution of vertebrate non-muscle actin genes. *Nucleic Acids Res* **14**: 5275–5294
Esch FS, Shimasaki S, Cooksey K, Mercado M, Mason AJ, Ying SY, Ueno N and Ling N (1987) Complementary deoxyribonucleic acid (cDNA) cloning and DNA sequence analysis of rat ovarian inhibins. *Mol Endocrinol* **1**: 388–396
Feijen A, Goumans MJ and Van Den Eijnden-Van Raaij AJM (1994) Expression of activin subunits, activin receptors and follistatin in postimplantation mouse embryos suggests specific developmental functions for different activins. *Development* **120**: 3621–3637
Feng ZM, Madigan MB and Chen CLC (1993) Expression of type II activin receptor genes in the male and female reproductive tissues of the rat. *Endocrinology* **132**: 2593–2600
Forman D and Møller H (1994) Testicular cancer. *Cancer Surv* **20**: 323–341
Giwercman A and Skakkebæk NE (1993) Carcinoma in situ of the testis: biology, screening and management. *Eur Urol* **2**: 19–21
Hashimoto M, Kondo S, Sakurai T, Etoh Y, Shibai H and Muramatsu M (1990) Activin/EDF as an inhibitor of neural differentiation. *Biochem Biophys Res Commun* **173**: 193–200
Hildén K, Tuuri T, Erämaa M and Ritvos O (1994) Expression of type II activin receptor genes during differentiation of human K562 cells and cDNA cloning of the human type IIB activin receptor. *Blood* **83**: 2163–2170
Hino M, Nishizawa Y, Tatsumi N, Tojo A and Morii H (1995) Down-modulation of c-kit mRNA and protein expression by erythroid differentiation factor/activin A. *FEBS Lett* **374**: 69–71
Hötten G, Neidhardt H, Schneider C and Pohl J (1995) Cloning of a new member of the TGF-β family: a putative new activin βC chain. *Biochem Biophys Res Commun* **206**: 608–613
Kaipia A, Parvinen M, Shimasaki S, Ling N and Toppari J (1991) Stage-specific cellular regulation of inhibin α-subunit mRNA expression in the rat seminiferous epithelium. *Mol Cell Endocrinol* **82**: 165–173
Kaipia A, Parvinen M and Toppari J (1993) Localization of activin receptor (ActR-IIB2) mRNA in the rat seminiferous epithelium. *Endocrinology* **132**: 477–479
Lau AL, Nishimori K and Matzuk MM (1996) Structural analysis of the mouse activin βC gene. *Biochim Biophys Acta* **1307**: 145–148
Ling N, Ying SY, Ueno N, Shimasaki S, Esch F, Hotta M and Guillemin R (1986) Pituitary FSH is released by a heterodimer of the β-subunits from the two forms of inhibin. *Nature* **321**: 779–782
Loveland KL, McFarlane JR and De Kretser DM (1996) Expression of activin βC subunit mRNA in reproductive tissues. *J Mol Endocrinol* **17**: 61–65
Martens JWM, De Winter JP, Timmerman MA, McLuskey A, Van Schaik RHN, Themmen APN and De Jong FH (1997) Inhibin interferes with activin signalling at the level of the activin receptor complex in CHO cells. *Endocrinology* **138**: 2928–2936
Mason AJ, Hayflick JS, Ling N, Esch F, Ueno N, Ying SY, Guillemin R, Niall H and Seeburg PH (1985) Complementary DNA sequences of ovarian follicular fluid inhibin show precursor structure and homology with transforming growth factor-β. *Nature* **318**: 659–663
Mason AJ, Niall HD and Seeburg PH (1986) Structure of two human ovarian inhibins. *Biochem Biophys Res Commun* **135**: 957–964
Mather JP, Attie KM, Woodruff TK, Rice GC and Phillips DM (1990) Activin stimulates spermatogonial proliferation in germ-Sertoli cell cocultures from immature rat testis. *Endocrinology* **127**: 3206–3214
Mather JP, Woodruff TK and Krummen LA (1992) Paracrine regulation of reproductive function by inhibin and activin. *Proc Soc Exp Biol Med* **201**: 1–15
Mathews LS (1994) Activin receptors and cellular signaling by the receptor serine kinase family. *Endocrinol Rev* **15**: 310–325
Mayo KE, Cerelli GM, Spiess J, Rivier J, Rosenfeld MG, Evans RM and Vale W (1986) Inhibin A-subunit cDNAs from porcine ovary and human placenta. *Proc Natl Acad Sci USA* **83**: 5849–5853
Michel U, Farnworth P and Findlay JK (1993) Follistatins: more than follicle-stimulating hormone suppressing proteins. *Mol Cell Endocrinol* **91**: 1–11
Mostofi FK (1980) Pathology of germ cell tumors of testis: a progress report. *Cancer* **45**: 1735–1754
Mostofi FK, Sesterhenn IA and Davis CJJ (1987) Immunopathology of germ cell tumors of the testis. *Semin Diagn Pathol* **4**: 320–341
Moul JW, McCarthy WF, Fernandez EB and Sesterhenn IA (1994) Percentage of embryonal carcinoma and of vascular invasion predicts pathological stage in clinical stage I nonseminomatous testicular cancer. *Cancer Res* **54**: 362–364
Müller J, Skakkebæk NE and Parkinson MC (1987) The spermatocytic seminoma: views on pathogenesis. *Int J Androl* **10**: 147–156
Oosterhuis JW, Castedo SMMJ, De Jong B, Cornelisse CJ, Dam A, Sleijfer DT and Schraffordt Koops H (1989) Ploidy of primary germ cell tumors of the testis. Pathogenetic and clinical relevance. *Lab Invest* **60**: 14–20
Oosterhuis JW and Looijenga LHJ (1993) The biology of human germ cell tumours: retrospective speculations and new prospectives. *Eur Urol* **23**: 245–250
Qu J and Thomas K (1995) Inhibin and activin production in human placenta. *Endocrinol Rev* **16**: 485–507
Sambrook J, Fritsch EF and Maniatis T (1989) *Molecular Cloning: a Laboratory Manual*. Cold Spring Harbor Press: New York
Shimasaki S, Koga M, Esch F, Cooksey K, Mercado M, Koba A, Ueno N, Ying SY, Ling N and Guillemin R (1988) Primary structure of the human follistatin precursor and its genomic organization. *Proc Natl Acad Sci USA* **85**: 4218–4222
Skakkebæk NE, Berthelsen JG, Giwercman A and Müller J (1987) Carcinoma-in-situ of the testis: possible origin from gonocytes and precursor of all types of germ cell tumours except spermatocytoma. *Int J Androl* **10**: 19–28
Strohmeyer T, Peter S, Hartmann M, Munemitsu S, Ackermann R, Ullrich A and Slamon DJ (1991) Expression of the hst-1 and c-kit protooncogenes in human testicular germ cell tumors. *Cancer Res* **51**: 1811–1816
Talerman A (1980) Spermatocytic seminoma: clinicopathological study of 22 cases. *Cancer* **45**: 2169–2176
Ten Dijke P, Ichijo H, Franzén P, Schulz P, Saras J, Toyoshima H, Heldin CH and Miyazono K (1993) Activin receptor-like kinases: a novel subclass of cell-surface receptors with predicted serine/threonine kinase activity. *Oncogene* **8**: 2879–2887
Ulbright TM (1993) Germ cell neoplasms of the testis. *Am J Surg Pathol* **17**: 1075–1091
Vale W, Rivier J, Vaughan J, McClintock R, Corrigan A, Woo W, Karr D and Spiess J (1986) Purification and characterization of an FSH releasing protein from porcine ovarian follicular fluid. *Nature* **321**: 776–779
Van Den Eijnden-Van Raaij AJM, Van Achterberg TAE, Van Der Kruijssen CMM, Piersma AH, Huylebroeck D, De Laat SW and Mummery CL (1991) Differentiation of aggregated murine P19 embryonal carcinoma cells is induced by a novel visceral endoderm-specific FGF-like factor and inhibited by activin A. *Mech Dev* **33**: 157–165
Van Dissel-Emiliani FMF, Grootenhuis AJ, De Jong FH and De Rooij DG (1989) Inhibin reduces spermatogonial numbers in testes of adult mice and Chinese hamsters. *Endocrinology* **125**: 1899–1903
Woodruff TK, Borree J, Attie KM, Cox ET, Rice GC and Mather JP (1992) Stage-specific binding of inhibin and activin to subpopulations of rat germ cells. *Endocrinology* **130**: 871–881
Wrana JL, Tran H, Attisano L, Arora K, Childs SR, Massagué J and O'Connor MB (1994) Two distinct transmembrane serine/threonine kinases from Drosophila melanogaster form an activin receptor complex. *Mol Cell Biol* **14**: 944–950

© Cancer Research Campaign 1997